# Tumor Suppressor Role of INPP4B in Chemoresistant Retinoblastoma

**DOI:** 10.1155/2023/2270097

**Published:** 2023-03-08

**Authors:** Natalia Miroschnikov, Oliver Dräger, Dario Van Meenen, Klaus Metz, Bettina Budeus, Nicole Dünker, Maike Anna Busch

**Affiliations:** ^1^Institute for Anatomy II, Department of Neuroanatomy, Center for Translational Neuro- and Behavioral Sciences (C-TNBS), University of Duisburg-Essen, Medical Faculty, Duisburg, Germany; ^2^Institute for Cellular Neurophysiology, University of Bielefeld, Medical Faculty, Bielefeld, Germany; ^3^Institute for Pathology, University of Duisburg-Essen, Medical Faculty, Duisburg, Germany; ^4^Institute for Cell Biology, University Hospital Essen, Essen, Germany

## Abstract

The chemotherapy of retinoblastoma (RB), a malignant ocular childhood disease, is often limited by the development of resistance against commonly used drugs. We identified inositol polyphosphate 4-phosphatase type II (INPP4B) as a differentially regulated gene in etoposide-resistant RB cell lines, potentially involved in the development of RB resistances. INPP4B is controversially discussed as a tumor suppressor and an oncogenic driver in various cancers, but its role in retinoblastoma in general and chemoresistant RB in particular is yet unknown. In the study presented, we investigated the expression of INPP4B in RB cell lines and patients and analyzed the effect of INPP4B overexpression on etoposide resistant RB cell growth *in vitro* and *in vivo*. INPP4B mRNA levels were significantly downregulated in RB cells lines compared to the healthy human retina, with even lower expression levels in etoposide-resistant compared to the sensitive cell lines. Besides, a significant increase in INPP4B expression was observed in chemotherapy-treated RB tumor patient samples compared to untreated tumors. INPP4B overexpression in etoposide-resistant RB cells resulted in a significant reduction in cell viability with reduced growth, proliferation, anchorage-independent growth, and *in ovo* tumor formation. Caspase-3/7-mediated apoptosis was concomitantly increased, suggesting a tumor suppressive role of INPP4B in chemoresistant RB cells. No changes in AKT signaling were discernible, but p-SGK3 levels increased following INPP4B overexpression, indicating a potential regulation of SGK3 signaling in etoposide-resistant RB cells. RNAseq analysis of INPP4B overexpressing, etoposide-resistant RB cell lines revealed differentially regulated genes involved in cancer progression, mirroring observed *in vitro* and *in vivo* effects of INPP4B overexpression and strengthening INPP4B's importance for cell growth control and tumorigenicity.

## 1. Introduction

Retinoblastoma (RB) is one of the most common malignant ocular diseases in early childhood worldwide [1, 2]. Various treatment options provide a patient survival rate of up to 95% [3, 4]. Intra-arterial, intravitreal, or intracameral drug injections significantly increase eye preservation rates and reduce systemic chemotherapy [5, 6]. Tumor treatment is, however, often limited by the massive side effects of the chemotherapeutics. Besides, the development of resistances against drugs of the commonly used VEC (vincristine, etoposide, and carboplatin) therapy ultimately lead to relapses or the emergence of secondary cancers [7]. Thus, new or adjacent RB therapy strategies are needed.

Our group identified inositol polyphosphate 4-phosphatase type II (INPP4B) as a downregulated gene in etoposide-resistant RB cell lines compared to their chemosensitive counterparts. Therefore, we assumed that INPP4B has a pivotal role in the development of RB resistance. A previous study by our group revealed that etoposide resistant RBs behave more aggressively than their chemosensitive counterparts. These cells display increased proliferation levels and, thus, higher growth kinetics *in vitro* and generate significantly more and larger tumors *in vivo* [8]. Other studies have already demonstrated that INPP4B is associated with chemoresistance in acute myeloid leukemia (AML; [9]) and induces chemosensitivity of human hepatocellular carcinoma cells lines [10].

The structure of INPP4B contains an N-terminal C2 lipid binding domain, an internal nervy homology 2 (NHR2) domain, and a C-terminal catalytic phosphatase domain, and it is one of a plethora of enzymes maintaining homeostasis of phosphoinositides within the cell [11]. A phosphoinositide (PI) is a membrane-bound inositol lipid that acts as a docking site for signaling proteins involved in proliferation, survival, and apoptosis. INPP4B has been reported as a negative regulator of the phosphatidylinositol kinase 3 (PI3K)/AKT signaling pathway [12, 13]. PI3K and phosphatidylinositol phosphate kinase 4 (PIP4K) phosphorylate phosphatidylinositol-3 (PI(3)P) and -4 phosphate (PI(4)P), which subsequently phosphorylate and thereby activate AKT, a potent driver of tumorigenic cell growth, which promotes cell proliferation, survival, and migration ([11, 14–16] for review see: [17]). Phosphorylated AKT in turn activates the signal transduction of other downstream molecules in the PI3K/AKT signaling pathway [18–20]. Chen et al. demonstrated that INPP4B overexpression in cervical carcinoma cells inhibits the activation of the PI3K pathway by suppressing the phosphorylation of AKT as well as serum and glucocorticoid-regulated kinase-3 (SGK3) [21]. SGK3, another PI3K-dependent serine/threonine kinase, displays high structural and functional similarities with the AKT protein [22].

Physiologically, INPP4B is highly expressed in epithelial cells of the breast and prostate glands, skeletal muscle, heart, brain, and pancreas [12]. *INPP4B* knockout (*Inpp4b*^−/−^) mice are viable and have a normal lifespan but develop defects in bone homeostasis at 8 weeks of age [23]. As INPP4B levels are significantly decreased in various cancers, it has first been described as a tumor suppressor gene, e.g., in prostate, basal-like breast, ovarian, cervical, gallbladder, and gastric cancer [21, 24–29], as well as thyroid neoplasm [30] and multiple myeloma [31]. However, several studies have reported an increased INPP4B expression, e.g., in AML, colon cancer, and a subset of melanomas [32–34] revealing the paradoxical role of an oncogene [17, 35].

The tumor suppressive function of INPP4B is most likely attributable to the negative regulation of PI3K/AKT signaling, whereas its oncogenic function is still unclear, and promotion of SGK3 signaling, inhibition of phosphatase and tensin homolog (PTEN)- dependent AKT activation, and enhancement of DNA repair mechanisms to confer chemoresistance have been proposed as potential mechanisms [17].

Thus, although its function in different human cancers remains controversial, INPP4B and the mediated PI3K/AKT downstream signaling pathway seem to play an important role in tumorigenesis and cancer progression. However, its biological role in retinoblastoma yet remain undiscovered. Thus, in the study presented, we set out to unravel the role of INPP4B in RB chemoresistance by analyzing INPP4B expression in etoposide resistant RB cell lines and chemotherapy-treated RB patient tumors and investigating the effect of lentiviral INPP4B overexpression on etoposide-resistant RB cell viability, proliferation, apoptosis, anchorage-independent growth, and tumor formation *in vitro* and *in vivo*. Besides, we studied the effect of INPP4B overexpression on the phosphorylation status of the known downstream signaling targets AKT and SGK3 and identified novel downstream signaling targets in a RNA sequencing analysis of INPP4B overexpressing, etoposide-resistant RB cell lines.

## 2. Material and Methods

### 2.1. Human Retina and Retinoblastoma Samples

For our comparative expression studies, we used tumor samples of retinoblastoma (RB) patients and postmortem healthy human retinae. The Ethics Committee of the Medical Faculty of the University of Duisburg-Essen approved the use of the above mentioned human samples (ethic approval #06-30214 and #14-5836-BO). Patients' parents or relatives gave their written informed consent to use the RB tumor samples.

### 2.2. Cell Lines and Culture

Dr. H. Stephan kindly provided the human RB cell line Y79 [36], originally purchased from the Leibniz Institute DSMZ (German Collection of Microorganisms and Cell Cultures) as well as the RB cell line RB355 [37], formerly donated by K. Heise, and the corresponding etoposide-resistant RB cell lines Y79-Etop and RB355-Etop. The cultivation of the above-mentioned cell lines as well as the human embryonic kidney cells (HEK293T) was described in detail previously [38]. No ethical approval was required for work with the human cell lines.

### 2.3. Lentiviral Expression Vector

To generate the INPP4B overexpression vector (pLenti_CMV_INPP4B), the human INPP4B cDNA sequence was cut from the pEAK-Flag/INPP4B plasmid (#24324, Addgene, Watertown, MA, USA; [27]) via XhoI and NotI fast digest restriction enzymes (Thermo Scientific, Oberhausen, Germany) and ligated into the XhoI and NotI digested pENTR4 vector (#17424; Addgene, Watertown, MA, USA; [39]). The gateway LR clonase II enzyme mix (Invitrogen, Darmstadt, Germany) was used to finally clone the full-length INPP4B sequence into the pLENTI_CMV_Puro_Dest vector (#17452; Addgene, Watertown, MA, USA; [39]) following the manufacturer's protocol. The empty pLenti_CMV_Puro_Dest vector served as a control vector in all INPP4B overexpression experiments.

### 2.4. INPP4B Overexpression

We generated lentiviral particles by transfecting 6 × 10^6^ HEK293 T cells with 6 *μ*g of each of the following plasmid DNAs, as described in detail previously [38]): (I) packaging vectors pczVSV-G [40] and pCD NL-BH [40], (II) pLenti_CMV_INPP4B for transduction of etoposide resistant Y79 and RB355 cells, (III) pLenti_CMV_Puro_Dest as a negative control for all overexpression experiments or (IV) GFP expression vector (pCL7EGwo) each together with 45 *μ*g polyethyleneimine (PEI, branched, Sigma-Aldrich, St. Louis, Missouri, USA) in DMEM medium. The medium was changed to Iscove's Modified Dulbecco's medium (IMDM, Pan-Biotech, Aidenbach, Germany) with 10% FCS and 1% penicillin/streptomycin after 24 h. 72 h later we harvested the viral supernatants, filtered and cryoconserved them.

For stable transduction, 1 × 10^6^/0.8 × 10^6^ RB cells (RB355/Y79) were seeded in DMEM medium as described previously [41]. The medium was removed after 24 h, and cells were transfected with INPP4B virus or control virus particles, and 5 *μ*L polybrene (H9268, Sigma-Aldrich, München, Germany) per ml lentivirus were administered. Twice the volume of the virus particles DMEM medium with supplements was added after 24 h. Forty-eight hours later we changed the medium completely and incubated the cells for another 72 h.

### 2.5. RNA Extraction and Quantitative Real-Time PCR

The NucleoSpin RNA II Kit (Macherey & Nagel, Düren, Germany) and the miRNeasy Kit (Qiagen, Hilden, Germany) were used for RNA isolations of RB cells. Complementary DNA for quantitative real-time PCR analyses was synthesized with the QuantiTect Reverse Transcription Kit (Qiagen, Hilden, Germany) following the manufacturer´s protocol. A SYBR™ green PCR assay (Applied Biosystem, Darmstadt, Germany) and the following specific primers were used to analyze INPP4B expression (see Table 1). GAPDH served as an endogenous control.

Real-time PCR reactions were conducted in duplicates in 20 *μ*L SYBR™ green PCR Mastermix (Applied Biosystem, Darmstadt, Germany) using the following program: 50°C for 2 min, 95°C for 2 min, 95°C for 30 sec and 60°C for 1 min (40 cycles), 95°C for 15 sec, 60°C for 30 sec, and 95°C for 15 sec.

For analyses of INPP4B expression in RB patient tumor samples, Hs01038078_m1 (INPP4B) and Hs03928990_g1 (18 S) TaqMan gene expression assays (Invitrogen, Darmstadt, Germany) were conducted in duplicate in 20 *μ*L TaqMan gene expression master mix (Invitrogen, Darmstadt, Germany) using the following program: 50°C for 2 min, 95°C for 10 min, 95°C for 15 sec, and 60°C for 1 min (40 cycles). Reactions were performed using a QuantStudio™ 3 real-time PCR system (ThermoFisher, Darmstadt, Germany).

### 2.6. RNA Seq Analysis

Concentration and quality of RNA were measured with Qubit (Invitrogen, Waltham, MA, USA) and Agilent Bioanalyzer DNA HS (Agilent, Santa Clara, CA, USA). Library preparation was performed with Lexogens QuantSeq 3′ mRNA-Seq Library Prep Kit FWD and sequenced on a NextSeq500 (Illumina, San Diego, CA, USA). Sequences were trimmed with TrimGalore (v.0.6.0 DOI: 10.14806/ej.17.1.200) and aligned with hisat2 (https://doi.org/10.1038/s41587-019-0201-4) to hg38. Statistical analysis was performed with R (R: A language and environment for statistical computing, R foundation for statistical computing, Vienna, Austria, version (v) 4.2.0 2022, https://www.R-project.org/) using the R-packages DESeq2 (10.1186/s13059-014-0550-8), pheatmap (v 1.0.12; Kolde; pheatmap: pretty heatmaps), umap (v 0.2.8.0; Konopka; umap: uniform manifold approximation and projection), fgsea (10.1101/060012) and EnhancedVolcano (v 1.14.0; Blighe, Rana, Lewis. EnhancedVolcano: Publication-ready volcano plots with enhanced colouring and labeling).

### 2.7. Western Blotting

Protein extraction and concentration measurements were described previously [42]. The same amounts of protein extracts were separated on a 10% SDS-PAGE and transferred onto nitrocellulose membranes. Membranes were incubated with primary antibodies against INPP4B (1 : 500; #PA5-58057; Invitrogen, Darmstadt, Germany), AKT (1 : 1,000; #4685; Cell Signaling Technology, Danvers, USA), p-AKT (1 : 500; #9271; Cell Signaling Technology, Danvers, USA), SGK (1 : 500; sc-166847; Santa Cruz, Dallas, USA), p-SGK (1 : 500; #5642; Cell Signaling Technology, Danvers, USA), and *β*-actin (1 : 1,000; #4967; Cell Signaling Technology, Danvers, USA) at 4°C overnight. HRP-conjugated species-specific secondary antibodies (goat-anti-rabbit; P0448 and rabbit-anti-mouse; P0260; DAKO, Glostrup, Denmark) were used in dilutions of 1 : 10,000 at room temperature for 1 hour. The signals were developed by the use of Western Bright Chemiluminescence Reagent (Cytiva, Buckinghamshire, UK).

### 2.8. Immunocytochemistry

For immunofluorescence staining, 1 × 10^5^ cells were seeded on poly-D-lysine (Sigma) coated coverslips and processed as described previously [41]. Primary antibody used: cleaved caspase-3 (1 : 400; Novo Castra; #9664 5A1E).

### 2.9. Cell Viability Assays

We determined cell viability by seeding 4 × 10^4^ cells in 100 *μ*L medium in a 96-well plate in two triplicates. Ten microliters of a water-soluble tetrazolium (WST-1) salt solution (Sigma-Aldrich, München, Germany) were added to each well after 72 h of incubation, and cells were incubated at 37°C for different time points. Quantification was performed in a microplate reader by measuring the absorbance at 450 nm.

### 2.10. Growth Kinetic

For growth kinetic analyses 3 × 10^5^ cells were seeded in triplicates in a 24-well plate in 500 *μ*L DMEM medium (PAN-Biotech, Aidenbach, Germany) with supplements. After trypan blue staining, vital cells were counted manually every 24 h (6 time points: 0 h, 24 h, 48 h, 72 h, 96 h, and 168 h) in a Neubauer chamber.

### 2.11. Cell Proliferation and Apoptosis Detection

Cell proliferation was determined by 5-Bromo-2′-deoxyuridine (BrdU; Sigma, Hamburg, Germany) incorporation as described previously [42].

### 2.12. Caspase-Glo 3/7 Assay

A caspase-Glo 3/7 assay (Promega, Madison, WI, USA) was used to analyze the caspase 3 and 7 cleavage activity after INPP4B overexpression, following the manufacturer´s instructions. Therefore, we seeded 9 × 10^4^ INPP4B overexpressing and control cells in 300 *μ*L growth medium supplemented with 2% FBS in a 24-well plate format and incubated them overnight. After 24 h, cell suspensions were mixed 1 : 1 with the caspase-3/7 reagent and seeded in a white 96-well plate for 2 h at room temperature protected from light. Luminescence was measured with an Orion II microplate luminometer (Berthold Detection Systems, Pforzheim, Germany). Measurements were performed three times in five replicates.

### 2.13. Colony Formation Soft Agarose Assay

Colony formation capacity was determined in soft agarose assays by suspending 5 × 10^3^ INPP4B overexpressing and control RB cells in 2 ml DMEM/F12 medium (Sigma-Aldrich, München, Germany) supplemented with 10% fetal bovine serum, 100 *μ* penicillin/ml and 100 *μ*g streptomycin/ml, 4 mM L-glutamine, 50 *μ*M *β*-mercaptoethanol, 10 *μ*g insulin/ml, and 0.7% agarose (Roth, Karlsruhe, Germany). Cell suspensions were layered on 2 ml 1% agarose as described previously [42] and maintained over a period of 3 weeks. Colony formation was quantified after 3 weeks of incubation, and assays were repeated three times. We determined the number of colonies for each cell line by counting colonies in eight visual fields at a 10x magnification in triplicates. A Nikon Eclipse TS2 microscope with a digital camera and IC measure 1.0 software (Nikon, Düsseldorf, Germany) was used to determine colony size and eight colonies per well were surveyed.

### 2.14. CAM Assays

The effects of INPP4B overexpression on tumor formation and migration capacity *in vivo* were studied in the chicken chorioallantoic membrane (CAM) assay. INPP4B overexpressing RB cells and control cells were inoculated on the CAM on embryonic development day (EDD) 10 mainly following the protocols published by [43, 44]. Ten eggs were inoculated with 1 × 10^6^ cells suspended in 50 *μ*L PBS in at least three independent experiments. At EDD17, grown tumors were excised, measured, weighted, and photographed as described previously [38]. Besides, GFP-labelled etoposide resistant Y79 and RB355 control and INPP4B overexpressing cells were injected into a CAM vene at EDD12 as described previously by our group [38]. Five days after injection, we sacrificed the chicken embryos and collected punches of the ventral CAM, opposing the injection site. The migrated, GFP-labelled cells were identified via fluorescence microscopy of the CAM punches.

Whole amount immunofluorescent staining of CAM vessels was described previously by our group [45]. A Nikon ECLIPSE E600 microscope and NIS Elements Imaging 5.20.02 software (Nikon, Düsseldorf, Germany) were used for imaging.

### 2.15. Statistical Analysis

We performed all assays at least in triplicates and used GraphPad Prism 4 for statistical analyses. The data represent means ± SEM of three independent experiments from independent RB cell cultures. The results were analyzed by a Student's *t*-test and considered significantly different if *p* value <0.05 (^*∗*^), *p* value <0.01 (^*∗∗*^), or *p* value <0.001 (^*∗∗∗*^). The growth curve statistics were performed using a free web interface https://bioinf.wehi.edu.au/software/compareCurves/, which uses the “compare growth curves” function from a statistical modeling package called statmod, available from the “R Project for Statistical Computing:” https://www.r-project.org, previously described elsewhere [46].

## 3. Results

### 3.1. INPP4B Is Differentially Expressed in Retinoblastoma Cell Lines and RB Patient Samples

**Figure 1:**
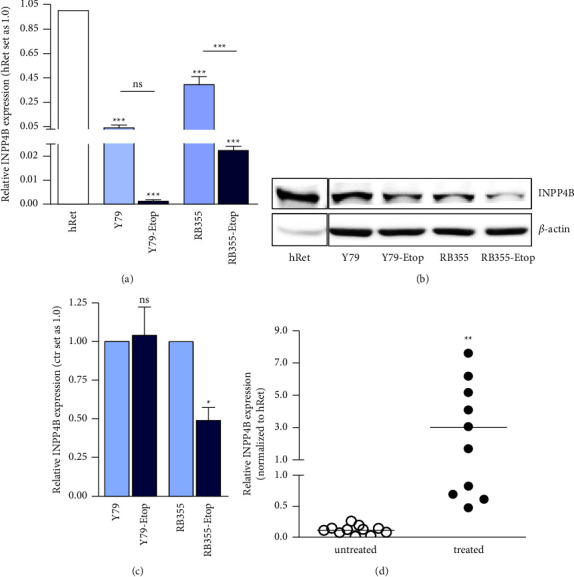
INPP4B expression in different retinoblastoma cell lines and primary RB patient tumors. The INPP4B expression in the healthy human retina (hRet) and chemosensitive as well as etoposide resistant (Etop) Y79 and RB355 retinoblastoma cell lines as revealed by quantitative real-time PCR (a) and western blot analyses (b). *ß*-Actin served as a loading control in (c) quantification of INPP4B protein expression in chemosensitive compared to etoposide resistant (Etop) Y79 and RB355 retinoblastoma cell lines. (d) INPP4B expression levels in primary RB patient tumors after treatment with chemotherapeutics (*n* = 10) and without treatment (*n* = 11). INPP4B expression values relative to expression in the healthy human retina and normalized to the expression of ribosomal 18S, used as an internal control. All experiments were performed at least in triplicate, and results were depicted with a standard error of the mean (SEM). A student's *t*-test was used to calculate statistical differences (ns *p* > 0.05; ^*∗*^*p* < 0.05; ^*∗∗*^*p* < 0.01; ^*∗∗∗*^*p* < 0.001) between control and experimental groups.

We analyzed the expression of INPP4B in parental, chemosensitive as well as etoposide-resistant Y79 RB suspension cells and in the adherent RB cell line RB355. Compared to the healthy human retina, on RNA level *INPP4B* was significantly downregulated in all RB cells lines investigated, with even lower expression levels in etoposide resistant compared to sensitive cell lines (Figure 1(a)). At the protein level, Western blot analysis confirmed a significantly lower INPP4B expression for etoposide-resistant RB355 cells compared to their chemosensitive counterparts (Figures 1(b) and 1(c)). In addition, a significant increase in *INPP4B* expression was observed in chemotherapy-treated RB tumor patient samples compared to untreated specimen (Figure 1(d)). The reduced expression in chemoresistant RB cells combined with increased expression levels in tumors of chemotherapy-treated RB patients potentially indicate an important role of INPP4B in the development of RB chemoresistance.

### 3.2. INPP4B Overexpression Reduces Cell Viability, Proliferation, and Growth of Etoposide Resistant Y79 and RB355 RB Cell Lines

**Figure 2:**
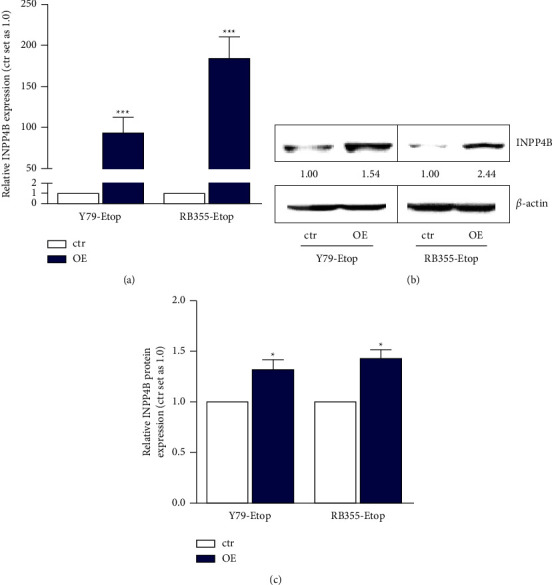
Verification of an efficient INPP4B overexpression (OE) in etoposide resistant Y79 and RB355 cells after lentiviral transduction as revealed by quantitative real-time PCR (a) and western blot analysis (b, c). *ß*-actin served as a loading control and micro manager 1.4 software was used to calculate the relative intensity ratios. All experiments were performed at least in triplicates and results depicted with a standard error of the mean (SEM). A student's *t*-test was used to calculate statistical differences (^*∗*^*p* < 0.05; ^*∗∗∗*^*p* < 0.001) between control and experimental groups.

We performed lentiviral INPP4B overexpression experiments in the etoposide resistant RB cell lines Y79 and RB355, both exhibiting significantly decreased INPP4B expression levels compared to the healthy human retina (Figures 1(a) and 1(b)). An efficient INPP4B overexpression was confirmed by quantitative Real-time PCR (Figure 2(a)) and Western blot analysis (Figures 2(b) and 2(c)).

**Figure 3:**
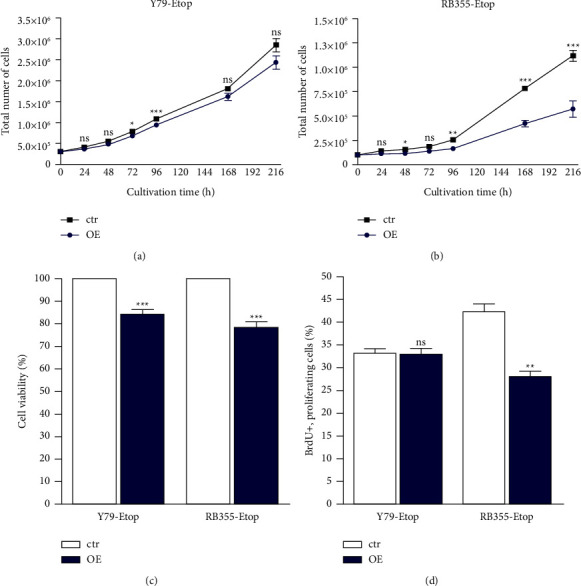
Effects of INPP4B overexpression in etoposide resistant (Etop) Y79 and RB355 cells. INPP4B overexpression (OE) significantly reduces cell growth of Y79-Etop (a) and RB355-Etop (b) cells with significant reduced cell viability for both RB cell lines and reduced proliferation level for RB355-Etop cells compared to control cells (ctr), as revealed by growth curves (a, b), WST-1 assays (c), and BrdU stains (d). All experiments were performed at least in triplicate, and results depicted with a standard error of the mean (SEM). A student's *t*-test was used to calculate statistical differences (ns *p* > 0.05; ^*∗*^*p* < 0.05; ^*∗∗*^*p* < 0.01; ^*∗∗∗*^*p* < 0.001) between control and experimental groups.

Compared to chemosensitive control cells, the INPP4B overexpressing etoposide-resistant RB cell lines investigated exhibited significantly decreased growth, lower cell viability, and decreased cell proliferation rates in RB355-Etop cells (Figure 3) as revealed by growth curve analyses (Figures 3(a) and 3(b)), WST-1 assays (Figure 3(c)) and BrdU cell counts (Figure 3(d)).

### 3.3. INPP4B Overexpression Induces Caspase Dependent Apoptosis in Etoposide Resistant Y79 and RB355 Cell Lines

**Figure 4:**
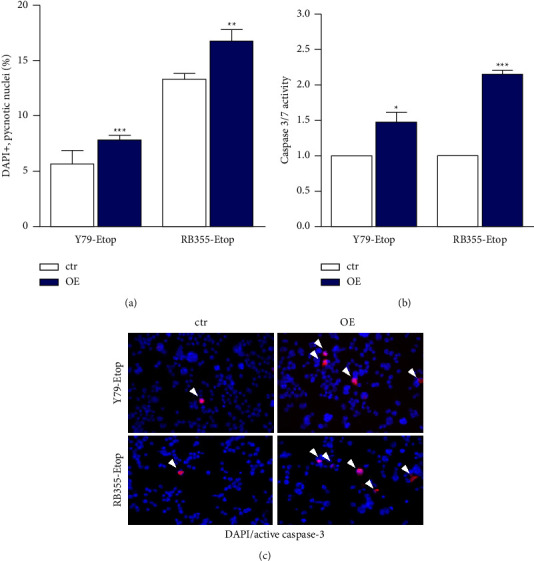
Effects of INPP4B overexpression on apoptosis levels in etoposide-resistant Y79 and RB355 cells. INPP4B overexpression (OE) increases the caspase-mediated apoptosis levels of etoposide-resistant (Etop) Y79 and RB355 cells compared to control cells (ctr) as revealed by DAPI cell counts (a) and caspase 3/7 assays (b). (c) Exemplary immunocytochemical stains of INPP4B overexpressing etoposide resistant Y79 and RB355 and control cells with an active, cleaved caspase-3 antibody (red fluorescence) and DAPI (blue fluorescence) counterstaining. White arrowheads indicate capase-3 positive cells. Magnification: 200x. All experiments were performed at least in triplicate and results were depicted with a standard error of the mean (SEM). A student's *t*-test was used to calculate statistical differences (^*∗*^*p* < 0.05; ^*∗∗*^*p* < 0.01; ^*∗∗∗*^*p* < 0.001) between control and experimental groups.

As revealed by DAPI cell counts, INPP4B overexpression resulted in a significant increase in apoptosis levels in etoposide resistant Y79 as well RB355 cell lines (Figure 4(a)). Caspase assays revealed that INPP4B likewise significantly increased caspase-3/7 activity in both RB cell lines investigated (Figure 4(b)). Additional immunocytochemical stains with an antibody against active, cleaved caspase-3 confirmed an increase in caspase-3 activity, indicated by a higher number of caspase-3 positive cells in INPP4B overexpressing cells. Together, these data indicate that INPP4B overexpression activates caspase-3/7 dependent apoptosis signaling.

### 3.4. INPP4B Overexpression Diminishes Anchorage-Independent Growth of Etoposide-Resistant Y79 and RB355 Cell Lines

**Figure 5:**
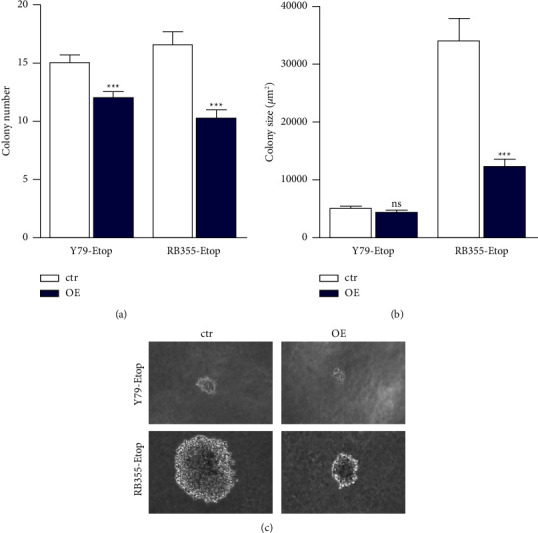
Effect of INPP4B overexpression on contact-independent growth of etoposide resistant Y79 and RB355 cells as revealed by soft agarose assays. Both RB cell lines display significantly reduced colony numbers (a), and RB355-Etop colonies were significantly smaller (b). (c) Representative photographs of colonies formed in soft agarose. All experiments were performed at least in triplicate and results depicted with a standard error of the mean (SEM). A student's *t*-test was used to calculate statistical differences (ns *p* > 0.05; ^*∗∗∗*^*p* < 0.001) between the control and experimental groups.

We next tested INPP4B overexpressing RB cells for alterations in anchorage-independent growth, known as the capacity of transformed, carcinogenic cells to grow without adherence to a solid surface [47]. Soft agarose assays revealed that both INPP4B overexpressing, etoposide resistant RB cell lines investigated form significantly fewer colonies than their parental, chemosensitive counterparts (Figure 5(a)). Besides, compared to control cell colonies, INPP4B overexpressing, etoposide resistant RB355 cell colonies were significantly smaller (Figures 5(b) and 5(c)).

### 3.5. INPP4B Overexpression Decreases Tumorigenicity of Etoposide Resistant RB Cells *In Vivo*

**Figure 6:**
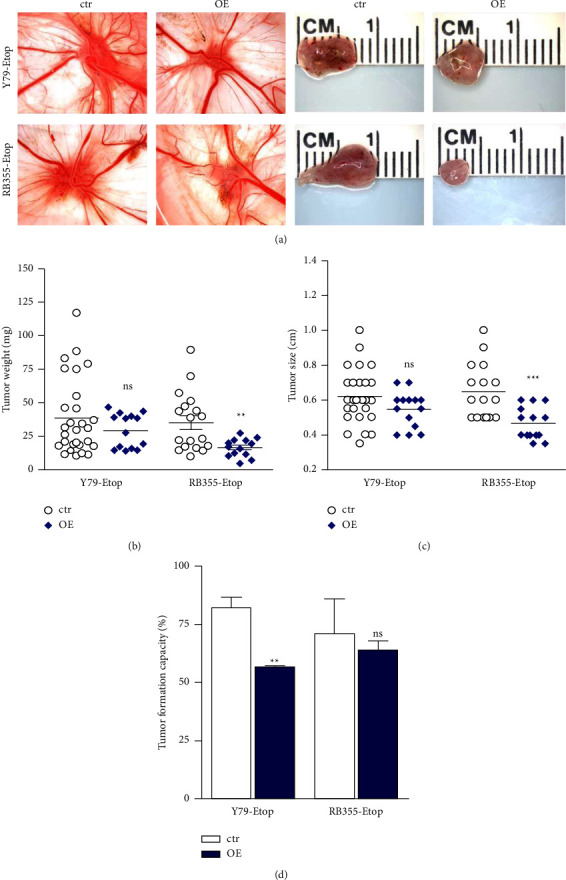
Impact of lentiviral INPP4B upregulation in etoposide resistant RB (etop) cells on tumor formation in CAM assays. (a) The left photo panel depicts CAM tumors *in ovo*, and the right panel measuring (in cm) of excised tumors. *In vivo* CAM assays revealed that tumors forming from INPP4B overexpressing (OE), etoposide resistant (Etop) Y79, and RB355 cells were smaller than those developing from control cells (ctr). (b–d) Graphical evaluation of CAM tumor weight (b), size (c), and tumor formation capacity (d). A student's *t*-test was used to calculate statistical differences (ns *p* > 0.05; ^*∗∗*^*p* < 0.01; ^*∗∗∗*^*p* < 0.001) between control and experimental groups.

To investigate whether INPP4B overexpression influences RB cells' tumor growth *in vivo*, we used the chicken chorioallantoic membrane (CAM) model.Etoposide-resistant, INPP4B overexpressing Y79 and RB355 cells and control cells were grafted onto the CAM of chicken embryos at embryonic day 10. Evaluation of CAM tumors developing from grafted RB cells (Figure 6(a)) as well as quantification of tumor weight (Figure 6(b)) and size (Figure 6(c)) showed that etoposide-resistant, INPP4B overexpressing RB355 cells developed significantly smaller tumors (Figures 6(a) and 6(b)) than control cells and likewise exhibited weight (Figure 6(b)). Inoculated etoposide resistant, INPP4B overexpressing Y79 cells did not generate significantly smaller tumors, but the tumor formation capacity was significantly decreased compared to chemosensitive control cells (Figure 6(d)).

### 3.6. INPP4B Overexpression Decreases Migration Potential of Etoposide-Resistant RB Cells *In Vivo*

**Figure 7:**
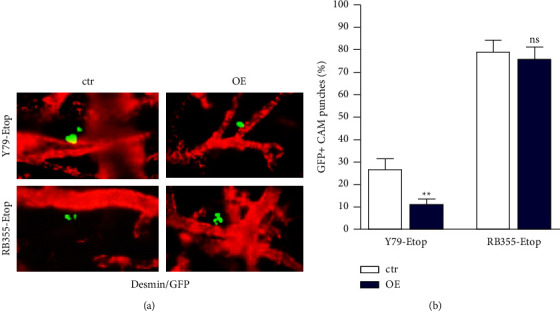
Depiction of the migratory potential of GFP-labelled, etoposide resistant (Etop) INPP4B overexpressing (OE) Y79 and RB355 cells and control cells (ctr) after injection into a CAM vein. (a) Representative CAM whole mounts stained for CAM vessels with an antidesmin antibody (red fluorescence). Extravasated GFP-labelled INPP4B overexpressing, etoposide resistant Y79 and RB355 cells are displayed in green. Magnification: 200x. (b) Quantification of GFP-positive (GFP+) punches of the lower CAM. All experiments were performed at least in triplicate and the results were depicted with a standard error of the mean (SEM). A student's *t*-test was used to calculate statistical differences (ns *p* > 0.05; ^*∗∗*^*p* < 0.01) between control and experimental groups.

Injection of GFP-labelled, etoposide-resistant Y79 and RB355 cells into an upper CAM vein led to extravasation of INPP4B overexpressing RB cells from the injection site into the CAM environment (Figure 7(a)). The cells migrated to a significant lesser extent than injected control cells (Figure 7(b)) as revealed by the quantification of GFP-positive, lower (opposite of the injection site) CAM punches. These results reveal that INPP4B overexpression results in a diminished tumorigenic and migratory potential *in vivo*.

### 3.7. INPP4B Overexpression Induces Phosphorylation of SGK3 but Did Not Affect AKT Signaling

**Figure 8:**
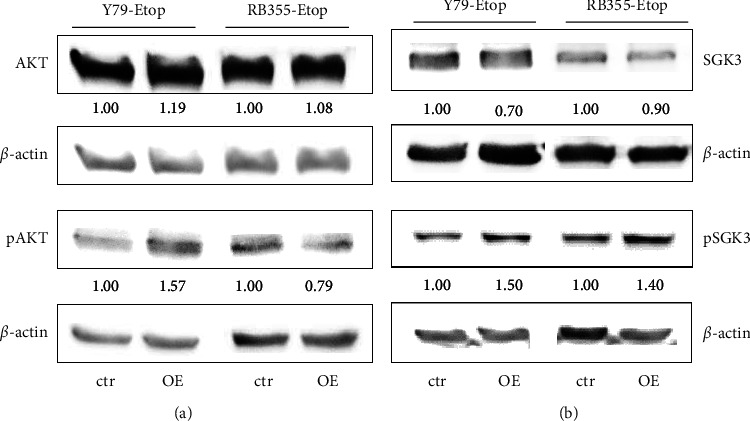
Western blot analyses of AKT/p-AKT and SGK3/p-SGK3 expression levels after INPP4B overexpression in etoposide resistant (Etop) RB cell lines. Representative western blots depicting AKT/p-AKT (a) and SGK3/p-SGK3 expression levels (b) after INPP4B overexpression (OE) in etoposide-resistant Y79 and RB355 cells. *ß*-actin served as a loading control and micro manager 1.4 software was used to calculate the relative intensity ratios.

AKT, a serine/threonine kinase, plays an important role in the PI3K signaling pathway and is known to be negatively regulated by INPP4B [13]. It has been shown that in cervical carcinoma cells INPP4B overexpression reduces the phosphorylation of AKT and SGK3, sharing structural and functional similarities [21]. Therefore, we investigated AKT, p-AKT, SGK3, and p-SGK3 expression after INPP4B overexpression in order to reveal the AKT and/or SGK3 pathways as potential INPP4B signaling mechanisms in etoposide-resistant RB cells. In both etoposide-resistant RB cell lines investigated, no significant changes in AKT levels were discernible following INPP4B overexpression and p-AKT expression was not consistently altered, being upregulated as well as downregulated in the respective cells (Figure 8(a) and Supplementary Figures 1(A) and 1(B). Total SGK3 levels were decreased, while p-SGK3 levels increased upon INPP4B overexpression (Figure 8(b)). Changes did not reach significance (Supplementary Figures 1(C) and 1(D), however, indicate that INPP4B potentially triggers the SGK3 signaling pathway in etoposide-resistant RB cells.

### 3.8. INPP4B Dependent Gene Regulation in Etoposide Resistant RB Cell Lines

**Figure 9:**
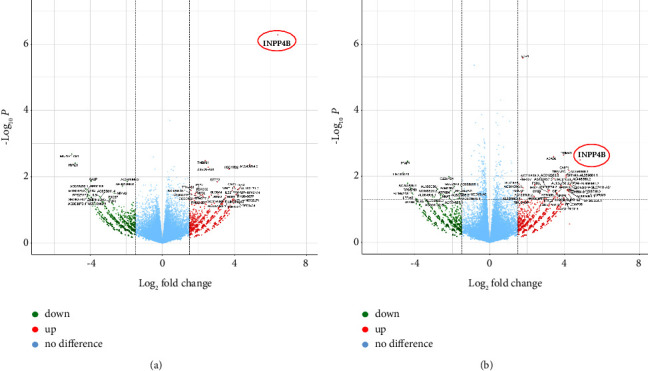
Volcano plots of differentially expressed genes (DEGs) after INPP4B overexpression in etoposide (Etop) resistant Y79 (a) and RB355 (b) cells compared to control cells. Downregulated DEGs are depicted in green, upregulated DEGs are depicted in red; nonregulated genes are depicted in blue. The red circle indicates INPP4B expression.

In order to investigate the molecular function of INPP4B in RB cells, we analyzed and compared gene expression profiles of INPP4B overexpressing etoposide resistant Y79 and RB355 with the respective control cells using RNAseq analysis. Volcano plot analysis shows the distribution of differentially expressed genes (DEGs) after INPP4B overexpression in Y79-Etop (Figure 9(a)), and RB355-Etop cells (Figure 9(b)), with INPP4B being identified as the most upregulated gene in both cell lines.

**Figure 10:**
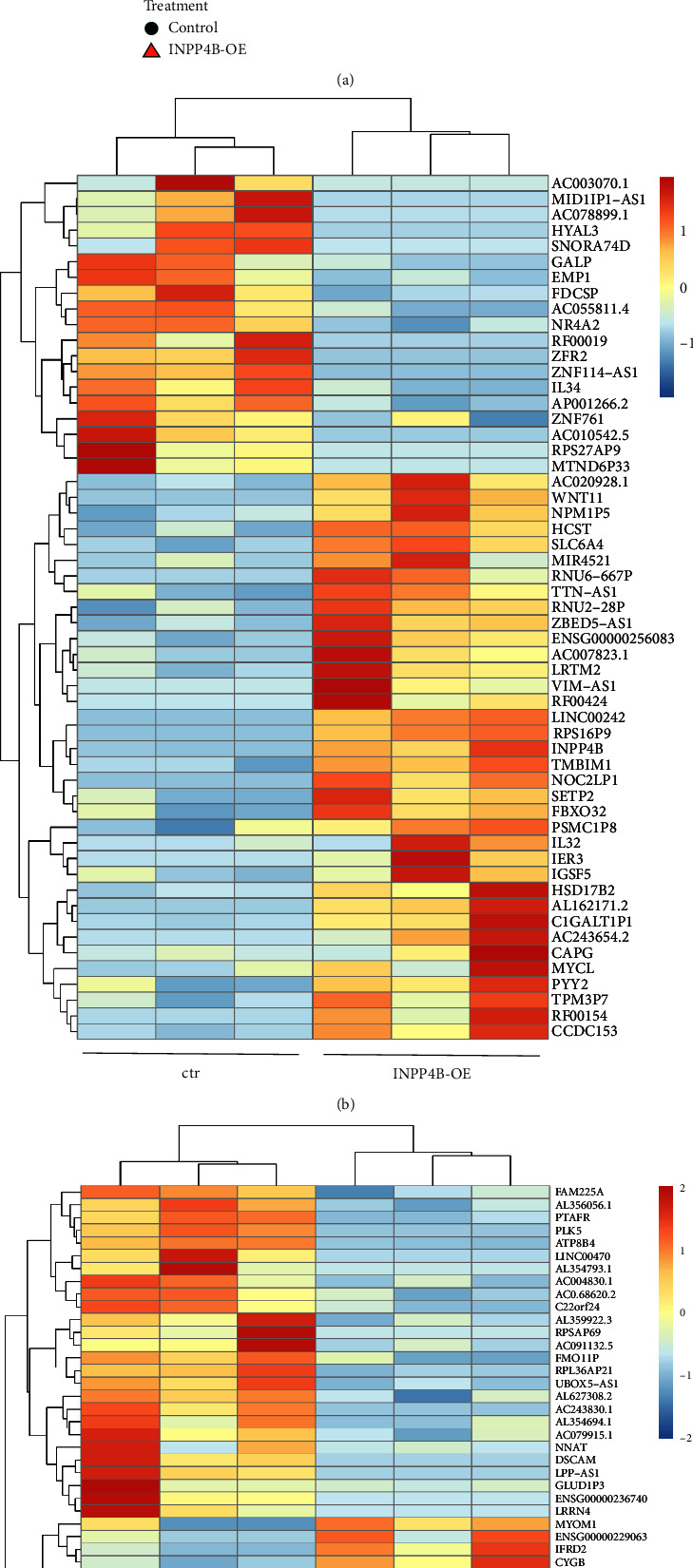
UMAP analysis and heatmap of the responsive gene selection (RGS) after INPP4B overexpression in etoposide (Etop) resistant RB cells and control cells. (a) UMAP analysis of three biological replicates of etoposide resistant Y79-Etop and RB355-Etop cells. (b) Heatmap of significantly up- and downregulated RGSs with a minimum fold chance of 1.5 after INPP4B overexpression in Y79-Etop (b) and RB355-Etop (c) cells.

UMAP analysis after INPP4B overexpression revealed a clear separation of an INPP4B overexpressing group compared to a control group in both etoposide-resistant RB cell lines investigated (Figure 10(a)). It became, however, evident, that the three biological replicates for each cell line themselves were different, most likely due to inner experimental variabilities (Figure 10(a)). Thus, subsequently, only genes with the same expression changes in all three biological replicates were included in further downstream analyses in order to exclude nonspecific gene expression changes.

To unravel INPP4B responsive genes, the mean of the three biological replicates was determined for each cell line and corresponding genes were selected (*p* < 0.05). Additionally, a minimum fold change (FC) of 1.5 relative to the controls were used as the criterion for an INPP4B responsive gene selection (RGS) cut off. A heatmap of the RGS for both cell lines analyzed is shown in Figures 10(b) and 10(c).

The RGS identified were used to perform pathway enrichment analysis in order to identify gene-related functions. DAVID analyses of differentially expressed genes filtered as RGS revealed four GO-terms with *p* < 0.05. Significantly related GO-terms were “positive regulation of gene expression” and “negative regulation of apoptotic signaling pathway” as well as “cytokine activity” for INPP4B overexpressing Y79-Etop cells and “cell adhesion” for INPP4B overexpressing RB355-Etop cells. No significant KEGG pathway connection could be identified for RGS of both RB cell lines investigated.

In order to narrow the list of DEGs, we subsequently reanalyzed the RGS and filtered only genes with a known gene function, excluding pseudogenes and noncoding RNAs. Thereupon, the number of genes significantly regulated after INPP4B overexpression was reduced to 16 upregulated and 8 downregulated genes for Y79-Etop cells (Table 2) and 25 upregulated and 6 downregulated genes for RB355-Etop cells (Table 3).

**Figure 11:**
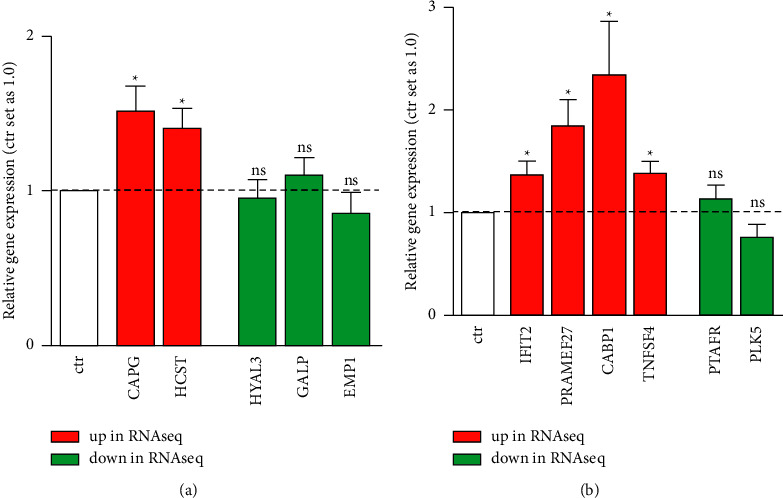
Validation of differentially expressed genes identified by RNAseq analysis after INPP4B overexpression in Y79-Etop (a) and RB355-Etop (b) RB cells. Quantitative real-time PCR verified the significant upregulation of *CAPG*, *HCST*, *IFIT2*, *PRAMEF27, CABP1*, and *TNFSF4* (red bars) after INPP4B overexpression. Downregulation of the genes *HYAL3, GALP, EMP1, PTAFR*, and *PLK5* did not reach significance (green bars). All experiments were performed at least in triplicates and results depicted with a standard error of the mean (SEM). A student's *t*-test was used to calculate statistical differences (^*∗*^*p* value <0.05) between control and experimental groups.

RNAseq data were validated for selected genes by quantitative real-time PCR, confirming that INPP4B overexpression causes a significant increase for all genes initially identified as upregulated (Figures 11(a) and 11(b), red bars). Besides, a slight yet not significant decrease in expression of *HYAL3* and *EMP1* in Y79-Etop (Figure 11(a)) and *PLK5* in RB355-Etop cells (Figure 11(b)) could be monitored following INPP4B overexpression. Downregulation of *GALP* (Figure 11(a)) and *PTAFR* (Figure 11(b)) could not be verified by Real-time PCR.

Our RNAseq data indicated that INPP4B overexpression in etoposide resistant RB cells led to several gene expression changes, potentially related to tumor progression. Especially the connected GO-Term “negative regulation of apoptotic signaling pathway” correlates well with the effects seen after INPP4B overexpression. Additionally, a subset of the highly significant regulated genes may be functionally involved in INPP4B-mediated effects on apoptosis and cell growth *in vitro* and therefore involved in etoposide-resistant RB tumor progression *in vivo*.

## 4. Discussion

INPP4B is a lipid phosphatase known to regulate phosphoinositide 3-kinase (PI3K)/AKT signaling. Originally, INPP4B was described as a tumor suppressor gene in various cancers, but it is now controversially discussed as an oncogenic driver (for review see: [11, 17]). However, increased INPP4B expression has been reported for several tumor entities, e.g., AML, melanoma, and colon cancers, suggesting the oncogenic potential of INPP4B (for review see: [17]).

Physiologically, INPP4B is highly expressed in human heart and skeletal muscle tissue [12]. Low INPP4B levels have been reported in various cancers and neoplasms [21, 24–27, 30], suggesting a tumor suppressor function in these tumor entities. INPP4B levels are significantly reduced in human hepatocellular, gastric, and gallbladder carcinoma [10, 28, 29]. In a most recent study, decreased INPP4B expression levels were reported for multiple myeloma cell lines as well as bone marrow plasma of multiple myeloma patients, and lower INPP4B levels correlated with a poor outcome [31]. Fittingly, in the study presented, we demonstrated that compared to the healthy human retina INPP4B mRNA expression levels are significantly decreased in RB cell lines, indicating a tumor-suppressing role of INPP4B in retinoblastoma. By contrast, INPP4B levels were upregulated in gallbladder and pancreatic cancer compared with non-tumor tissues [29, 48], suggesting an oncogenic role in these tumor entities. Increased INPP4B expression was likewise reported in acute myeloid leukemia, colon cancer, and some melanoma subtypes [32–34] supporting the notion of INPP4B as an oncogene [17, 35]. Moreover, high INPP4B expression has been reported in gastric cancer patients with large tumors and low to undifferentiated metastasis, which is correlated with a poor prognosis. By contrast, INPP4B expression correlated with a good prognosis in patient with small tumors in a highly to moderately differentiated metastasis stage [28]. Further along this line, primary nonmetastatic colorectal cancer stem-like cells (CR-CSLCs) display significantly reduced INPP4B levels, while they are increased in highly metastatic CR-CSLCs [49]. Based on these data, it has been hypothesized that *INPP4B* may have seemingly contradictory functions as an oncogenic driver or a tumor suppressor depending on the tumor entity, cancer grade, and clinical stage [29, 49].

It has been demonstrated that overexpression of INPP4B induces chemosensitivity in human hepatocellular carcinoma and prostate cancer cells lines [10, 50]. Wang et al. likewise demonstrated that INPP4B overexpressing multiple myeloma cells become more sensitive to bortezomib, while INPP4B knockdown cells became more resistant to bortezomib treatment strongly suggesting INPP4B as a key regulator of chemosensitivity [31]. Accordingly, INPP4B overexpression inhibited chemoresistance of primary nonmetastatic CR-CSLCs, but increased chemosensitivity in metastatic CR-CSLCs [49]. In our study, decreased INPP4B expression levels in etoposide resistant compared to chemosensitive RB cell lines and increased levels in chemotherapy-treated RB patient tumors compared to the nontreated likewise strongly suggested an impact of INPP4B on the etiology of etoposide chemoresistance in RB.

In the study presented, INPP4B overexpression reduced proliferation, viability, and growth of etoposide-resistant RB cell lines and concomitantly increased caspase-3/7 mediated apoptosis levels, supporting INPP4B`s role as a tumor suppressor in RB cells. Consistent with our data, it has been demonstrated that INPP4B overexpression inhibits cervical and human hepatocellular carcinoma (HCC) as well as multiple myeloma and acute myeloid leukemia, cell proliferation, and induces caspase-3-mediated apoptosis in HCC cell lines [10, 21, 31]. Similarly, INPP4B knockdown increased the proliferation of human basal-like breast cancer cells [26]. By contrast, INPP4B downregulation reduced proliferation and increased apoptosis of gastric, pancreatic, and gallbladder cancer cells, while INPP4B overexpression leads to opposing effects [28, 29, 48]. Further along this line, loss of INPP4B significantly inhibited proliferation of NPM1-mutated OCI-AML3 cells [51], and overexpression of INPP4B enhanced proliferation of melanoma cells and melanocytes as well as colon cancer cells, in which INPP4B acts as an oncogenic driver [33, 34].

We demonstrated that INPP4B overexpression reduces anchorage-independent growth of etoposide-resistant RB cell lines, reflecting its tumor suppressive function. Fittingly, INPP4B overexpression inhibits colony formation and anchorage-independent growth of human hepatocellular and cervical carcinoma cells lines [10, 21] as well as induced expression of INPP4B in human breast cancer cells without INPP4B expression reduced anchorage-independent growth [26]. Similarly, knockdown of INPP4B in thyroid, mammary epithelial cell and breast cancer cell lines provided an advantage for anchorage-independent growth [26, 27, 30]. By contrast, INPP4B overexpression in acute myeloid leukemia cells increased their colony formation potential [32], and INPP4B overexpression likewise led to enhanced anchorage-independent growth in cancer entities, in which INPP4B was identified as an oncogene, e.g., gallbladder cancer, colon cancer, and acute myeloid leukemia cell lines [29, 34, 51].

In the study presented INPP4B overexpression in etoposide-resistant RB cells resulted in decreased tumor formation capacity or reduced size of CAM tumors *in ovo.* Besides, the migration potential was decreased at least in one RB cell line investigated, strengthening the anti-tumorigenic role of INPP4B in retinoblastoma. Fittingly, INPP4B overexpression in cervical cancer and ductal carcinoma cells decreased tumor growth in mice [21, 27], and INPP4B knockdown in breast cancer cells increased the number and size of tumors in an athymic murine xenograft model [26]. Moreover, in a genetically-engineered triple-negative breast cancer mouse model INPP4B knockout mice displayed a significant, dose-dependent increase in tumor emergence, indicating a tumor suppressor function of INPP4B in these tumor entities [25]. By contrast, INPP4B depletion in melanocytes leads to a delay in tumor development *in vivo,* suggesting a tumorigenic capacity of INPP4B in this setting [33].

INPP4B has been reported as a negative regulator of PI3K/AKT signaling [13] and was anticipated to act as a tumor suppressor by inhibiting this pathway [27]. Most recently, Wang et al. demonstrated that INPP4B overexpression decreased the phosphorylation of AKT in multiple myeloma and hepatocellular carcinoma cells [31], whereas tumors derived from INPP4B knockout mice were found to be enriched for AKT [25]. It has, however, been shown that in cervical carcinoma cells INPP4B overexpression reduces the phosphorylation of both AKT and SGK3, sharing structural and functional similarities [21]. Therefore, we investigated the phosphorylation status of AKT and SGK3 following INPP4B overexpression in order to reveal the possible involvement of each pathway as potential INPP4B signaling mechanisms in etoposide resistant RB cells. No significant changes in AKT or p-AKT levels were discernible following INPP4B overexpression, whereas p-SGK3 levels increased, indicating a potential involvement of the SGK3 signaling pathway in RB etoposide-resistance. In accordance with our data, INPP4B overexpression promoted SGK3 phosphorylation but did not influence p-AKT levels in AGS gastric cancer cells, while INPP4B reduction elevated AKT phosphorylation, but did not increase p-SGK3 levels in BGC823 gastric cancer cells [28]. Along this line, INPP4B knockdown leads to a reduction in p-SGK3 levels, but did not influence AKT activation in NPM1-mutated OCI-AML3 acute myeloid leukemia cells [51] and INPP4B expression is not correlated with alterations in AKT phosphorylation in leukemia, suggesting an AKT-independent mechanism [9]. Indeed, INPP4B seems to alternatively signal via SGK3 in cells without canonical AKT signaling [52]. Studies indicated a correlation between high INPP4B expression and SGK3 phosphorylation levels in breast cancer and melanoma cells, in which INPP4B overexpression triggered phosphorylation and activation of SGK3, not AKT. In these cancer cell lines INPP4B signaling, however, increased proliferation and anchorage-independent growth [33, 53]. Thus, recent studies reporting on INPP4B-mediated activation of SGK3 depict INPP4B as an oncogenic driver [9, 28, 33, 34], whereas our functional studies indicate a tumor suppressive role of INPP4B in retinoblastoma.

INPP4B overexpression in etoposide-resistant RB cells induced changes in gene regulation revealed by RNAseq analysis. GO-term analysis of the RGS revealed connections to apoptosis (GO-term “negative regulation of apoptotic signaling pathway”) and gene expression regulation (GO-term “gene expression regulation”) as well as cytokine activity and cell adhesion. These data are in line with the results of our *in vitro* experiments which showed induced caspase dependent apoptosis levels following INPP4B overexpression in both etoposide-resistant cell lines investigated. A main difference between the two RB cells lines investigated is their growth behavior and fittingly, the significant GO-term “cell adhesion” is connected to RB355 cells, growing as an adherent culture. Tumor cell adhesion to the extracellular matrix is an important facilitator of therapy resistance. It could be shown that the cell adhesion resistome is involved in the homeostasis of cancer cells and fundamentally contributes to adaptation mechanisms, including survival and growth, induced by molecular drugs [54]. The observation that different DEGs have been identified for the two RB cell lines investigated is most likely attributable to the fact that Y79 and RB355 retinoblastoma cells are different *per se*, one growing as suspension cells (Y79) and one, as mentioned above, as an adherent culture (RB355).

Interestingly, some of the verified upregulated genes of the identified RGS seem to trigger the oncogenic role of INPP4B, which is not reflected by our functional data. However, mirroring previously described effects of an increased phosphorylation of SGK3 seen after INPP4B overexpression in both RB cell lines. In this context, INPP4B has already been described as an oncogenic driver through phosphorylation and activation of SGK3 in a subset of melanoma and colon carcinoma [33, 34].

One of the upregulated genes potentially driving the oncogenic role of INPP4B in RB is the macrophage-capping protein (CAPG) which raised the expression of CAPG was likewise shown in different metastatic cancers, supporting its involvement in tumor cell invasion and metastatic processes [55, 56]. Increased CAPG expression has been correlated with elevated invasiveness and migration in several human tumor entities like, e.g., glioblastoma [57]. Besides, increased CAPG expression strongly correlates with the resistance to paclitaxel chemotherapy [58], and knockdown of the circular RNA circ_0055412 promotes the cisplatin sensitivity of glioma cells through modulation of the CAPG signaling pathway [59]. Finally, elevated CAPG expression is correlated with unfavorable clinical parameters and poor patients` outcomes in different cancers, suggesting a potential role as a biomarker for prognosis and prediction of therapy outcome [60–62].

The hematopoietic cell signal transducer HCST, also upregulated in INPP4B overexpressing etoposide resistant Y79 RB cells, has been suggested as a potential biomarker for renal cell carcinoma and lung cancer diagnosis and prognosis [63, 64]. High HCST expression leads to significant enrichment in cell adhesion, tumor formation, and immune and inflammatory responses in a renal cell carcinoma specimen [63].

Additionally, we identified an upregulation of the tumor necrosis factor superfamily member 4 (TNFSF4) after INPP4B overexpression in etoposide-resistant RB355 cells. Higher levels of TNFSF4 were likewise detected in breast and bladder carcinoma as well as in serum and tumor tissues of lung adenocarcinoma patients, and it has been shown that stress-induced induction of TNFSF4 in cancer-associated fibroblasts alleviates the resistance of lung adenocarcinomas against chemotherapeutics by inhibiting tumor cell apoptosis [65–67].

High levels of the calcium-binding protein 1 (CABP1), as detected after INPP4B overexpression in etoposide-resistant RB355 cells, were likewise revealed by Kaplan–Meier analysis of glioblastoma hub genes and was negatively associated with relapse-free survival of glioblastoma patients [68]. Besides, *CABP1* was identified as one of 5 key prognostic genes for predicting the survival of invasive lobular breast cancer survival [69]. Additionally, CABP1 can adjust the activity of inositol 1,4,5-triphosphate receptors in a calcium-dependent manner [70]. Regardless of the described functions, up to now CABP1 has seldom been investigated in the context of oncological research.

Functions mediated by the verified upregulated interferon-induced protein with tetratricopeptide repeats (IFIT) and the downregulated epithelial membrane protein 1 (EMP1) are in line with the tumor suppressive function of INPP4B seen in RB cells. Members of the IFIT genes have been shown to promote drug resistance after depletion and are negatively associated with tumor malignancy due to proapoptotic effects and the activation of caspase-3 after overexpression (for review see [71]). Besides, their potential use as cancer biomarkers and prognostic factors as well as novel therapeutic targets for cancer therapy has been discussed (for review see [71, 72]. EMP1 is a transmembrane glycoprotein involved in oncogenic processes like proliferation, migration, invasion, metastasis, and malignant progression [73–75]. In a previous study by our group, we demonstrated that EMP1 knockdown in RB cells significantly reduces cell viability and proliferation and increases apoptosis [76]. Real-time PCR verification of our RNAseq data, revealing a downregulation of EMP1 in INPP4B overexpressing, etoposide-resistant Y79 cells, did, however, not reach significance.

Nevertheless, further functional experiments will be required to unravel the interplay between INPP4B and the identified differentially expressed genes in terms of tumor suppressive as well as oncogene-like impacts.

## Figures and Tables

**Table 1:** Primer sequences for the genes analyzed by real-time PCR analysis. FW: forward primer; RV: reverse primer.

Genes	Primer sequences
CAPG-FW	5′-AGGAGCCTGCTGAGATGATC-3′
CAPG-RV	5′-AGCAGTTCAAGGGCAAATGG-3
HCST-FW	5′-GGTCACATCCTCTTCCTGCT-3′
HCST-RV	5′-CATCTTCTTGGGCGGGGC-3′
HYAL3-FW	5′-CTTCCCCAGCATCTACCTCC-3′
HYAL3-RV	5′-CACACCAATGGACTGCACAA-3′
GALP-FW	5′-TGGACCCTCAATAGTGCTGG-3′
GALP-RV	5′-AGCATGCCCAGATCTCCAAT-3′
EMP1-FW	5′-TTCTGTGTCATTGCCCTCCT-3′
EMP1-RV	5′-GACCAGATAGAGAACGCCGA-3′
IFIT2-FW	5′-ACCTGGAACTTGATGGAGGG-3′
IFIT2-RV	5′-AGACCCAGGCATAGTTTCCC-3′
PRAMEF27-FW	5′-TTCCCCAGAGCAGAAGAAGG-3′
PRAMEF27-RV	5′-CACTCAGGTCCAGGGTCTTT-3′
CABP1-FW	5′-GTGGAGCTAATGGGGCCTAA-3′
CABP1-RV	5′-CCTCTATGTCTCGGTGTCCC-3′
TNFSF4-FW	5′-ACCTACATCTGCCTGCACTT-3′
TNFSF4-RV	5′-TGACTGAGTTGTTCTGCACC-3′
PTAFR-FW	5′-GGACAGCAAATTCCACCAGG-3′
PTAFR-RV	5′-AGGGATCTGGTTGAATGGCA-3′
PLK5-FW	5′-ACGACTTCTTCACACAGGGT-3′
PLK5-RV	5′-GGACCCGAGGCCTCTTTAG-3′
INPP4B-FW	5′- CACTGGTGCAGATCTCCGTA -3′
INPP4B-RV	5′- CAAAAACAGTGGGTCCCTTG -3′
GAPDH-FW	5′-ACCCACTCCTCCACCTTTGA-3′
GAPDH-RV	5′-CTGTTGCTGTAGCCAAATTCGT-3′

**Table 2:** Responsive gene selection (RGS) after INPP4B overexpression in etoposide-resistant Y79 cells. Genes with positive fold change (FC) values represent upregulated genes after INPP4B overexpression, while negative FC values indicate downregulated genes.

*Gene symbols*	*Description of upregulated DEGs*	*FC*	*P-value*
*INPP4B*	Tumor suppressor; involved in phosphatidylinositol signaling	6.41	0.000
*IER3*	Protects cells from fas or tumor necrosis factor type alpha induced apoptosis	4.30	0.021
*WNT11*	Implicated in oncogenesis and several developmental processes; member of the WNT gene family	4.20	0.025
*CAPG*	Reversibly blocks the barbed ends of F-actin filaments in a Ca2+ and phosphoinositide-regulated manner	4.00	0.021
*RF00424*	Small cajal body-specific RNA 16	3.97	0.047
*IL32*	Encodes a member of the cytokine family	3.87	0.026
*HSD17B2*	Enables estradiol 17-beta-dehydrogenase and testosterone dehydrogenase (NAD+) activity; involved in response to retinoic acid	3.69	0.006
*HCST*	Activates phosphatidylinositol 3-kinase dependent signaling pathways; receptor complex may have a role in cell survival and proliferation	3.52	0.037
*MIR4521*	Reduces proliferation and invasion of medulloblastoma cells; induces programmed cell death via activation of caspase 3/7	3.51	0.044
*SLC6A4*	Encodes an integral membrane protein that transports serotonin from synaptic spaces into presynaptic neurons	3.09	0.034
*LRTM2*	Predicted to be involved in axon guidance and negative chemotaxis	2.71	0.035
*TMBIM1*	Negative regulator of extrinsic apoptotic signaling pathway via death domain receptors	2.40	0.003
*CCDC153*	Protein coding; enables identical protein binding activity	2.22	0.036
*MYCL*	Predicted to enable DNA-binding transcription factor activity	2.12	0.035
*FBXO32*	Encodes a member of the F-box protein family	1.89	0.030
*IGSF5*	Predicted to enable PDZ domain binding activity and cell-cell adhesion	1.71	0.047

*Gene symbols*	*Description of downregulated DEGs*	*FC*	*P-value*
*HYAL3*	Regulates turnover of hyaluronan, which plays a critical role in biological like cell proliferation, migration and differentiation	−4.82	0.004
*ZFR2*	Predicted to enable single- and double-stranded RNA binding activity	−4.09	0.031
*GALP*	Encodes a member of the galanin family of neuro-peptides; may serve as a marker for neuroblastic tumors	−3.96	0.014
*IL34*	Cytokine that promotes the differentiation and viability of monocytes and macrophages through colony-stimulating factor-1 receptor	−3.49	0.040
*EMP1*	Potential biomarker for tumor diagnosis and prognosis of several cancers	−3.45	0.045
*FDCSP*	Potential contribution to tumor metastases by promoting cancer cell migration and invasion	−2.92	0.048
*NR4A2*	Encoded protein may act as a transcription factor	−2.67	0.032
*ZNF761*	Predicted to enable DNA-binding transcription factor activity	−1.46	0.046

**Table 3:** Responsive gene selection (RGS) after INPP4B overexpression in etoposide resistant RB355 cells. Genes with positive fold change (FC) value represent upregulated genes after INPP4B overexpression, while negative FC values indicate downregulated genes.

*Gene symbols*	*Description of upregulated DEGs*	*FC*	*P-value*
*INPP4B*	Tumor suppressor; involved in phosphatidylinositol signaling	6.26	0.000
*CNTNAP5*	Contactin associated protein family member 5	4.83	0.003
*VSIG8*	V-set and immunoglobulin domain containing 8	4.32	0.027
*CD44*	Involved in cell-cell interactions, cell adhesion and migration; receptor for hyaluronic acid; possibly related to tumor metastasis	4.29	0.018
*PRAMEF27*	Predicted to be involved in negative regulation of apoptotic process; negative regulation of transcription and positive regulation of proliferation	4.23	0.029
*CABP1*	Regulates calcium-dependent activity of inositol 1,4,5-triphosphate receptors; predominantly expressed in retina and brain	4.23	0.007
*GLIPR1L2*	Members of this family have roles in a variety of processes, including cancer and immune defense	4.21	0.035
*GPSM3*	Predicted to enable GTPase regulator activity and to be involved in positive regulation of inflammatory response	4.16	0.026
*MYH8*	Encodes a member of the class II or conventional myosin heavy chains; functions in skeletal muscle contraction	3.97	0.050
*TRIM49*	Contains a RING zinc finger, a motif known to be involved in protein-protein interactions	3.94	0.002
*TRIM49 C*	Predicted to enable ubiquitin protein ligase activity	3.62	0.008
*TNFSF4*	Encodes a cytokine of the tumor necrosis factor (TNF) ligand family	3.57	0.033
*GIF*	Member of the cobalamin transport protein family, glycoprotein secreted by parietal cells of the gastric mucosa	3.56	0.034
*ACADL*	Belongs to the acyl-CoA dehydrogenase family	3.41	0.003
*FGD5*	Predicted to enable guanyl-nucleotide exchange factor activity and small GTPase binding activity	3.40	0.048
*IFIT2*	Enables RNA binding activity; involved in positive regulation of apoptotic process	3.12	0.030
*WFIKKN2*	Contains a WAP, follistatin, and immunoglobulin domain, two tandem kunitz domains, and a NTR domain	2.92	0.047
*FSD2*	Encodes a protein that belongs to the FN3/SPRY family	2.79	0.025
*CYGB*	may be involved in protection during oxidative stress	2.68	0.034
*SLITRK1*	Thought to be involved in neurite outgrowth	2.40	0.019
*ETV4*	Involved in positive regulation of keratinocyte differentiation and transcription by RNA polymerase II	1.76	0.000
*RNF227*	Predicted to enable metal ion binding activity	1.71	0.011
*MYOM1*	Myomesin 1 and other myofibrillar proteins contain structural modules with strong homology to fibronectin type III (motif I) or immunoglobulin C2 (motif II) domains	1.70	0.032
*SLC16A8*	Member of a family of proton-coupled monocarboxylate transporters mediating lactate transport across cell membranes	1.58	0.017
*COL7A1*	Functions as an anchoring fibril between external epithelia and underlying stroma	1.50	0.017

*Gene symbols*	*Description of downregulated DEGs*	*FC*	*P-value*
*PTAFR*	G-protein-coupled receptor for platelet-activating factor (PAF); stimulates signal transduction pathways including the phosphatidylinositol-calcium second messenger system	−4.39	0.003
*DSCAM*	Involved in human central and peripheral nervous system development	−4.23	0.023
*LRRN4*	Predicted to act upstream of or within visual learning	−3.94	0.050
*PLK5*	Involved in defense response to tumor cells; positive regulation of neuron projection development	−3.90	0.050
*ATP8B4*	Involved in phospholipid transport in the cell membrane	−2.62	0.036
*NNAT*	Possibly involved in the regulation of ion channels during brain development; role in forming and maintaining nervous system structure	−2.49	0.036

## Data Availability

The RNAseq data used to support the findings of this study have been deposited in the Gene Expression Omnibus (GEO) database (GSE226408).

## Supplementary Materials

Supplementary Materials Supplementary Figure 1: Quantification of Western blot analyses of AKT/p-AKT (A+B) and SGK3/p-SGK3 (C+D) expression levels after INPP4B overexpression in etoposide resistant (Etop) RB cell lines. *ß*-actin was used as a loading control. Values are means of at least three independent experiments ± SEM. ns *p* > 0.05 statistical differences compared to the control group calculated by Students *t*-test.

## References

[1] Bornfeld N., Lohmann D., Bechrakis N. E., Biewald E. (2020). Retinoblastom. *Ophthalmologe, Der: Zeitschrift der Deutschen Ophthalmologischen Gesellschaft*.

[2] Fabian I. D., Khetan V., Stacey A. W. (2022). Sex, gender, and retinoblastoma: analysis of 4351 patients from 153 countries. *Eye*.

[3] Dimaras H., Corson T. W. (2018). Retinoblastoma, the visible CNS tumor: a review. *Journal of Neuroscience Research*.

[4] Meel R., Kashyap S., Bakhshi S., Singh Bajaj M., Wadhwani M. (2020). Retinoblastoma in children older than 6 Years of age. *Ocular oncology and pathology*.

[5] Kaewkhaw R., Rojanaporn D. R. (2020). Retinoblastoma: etiology, modeling, and treatment. *Cancers*.

[6] Munier F. L., Beck-Popovic M., Chantada G. L. (2019). Conservative management of retinoblastoma: challenging orthodoxy without compromising the state of metastatic grace “Alive, with good vision and no comorbidity”. *Progress in Retinal and Eye Research*.

[7] Temming P., Arendt M., Viehmann A. (2017). Incidence of second cancers after radiotherapy and systemic chemotherapy in heritable retinoblastoma survivors: a report from the German reference center. *Pediatric Blood and Cancer*.

[8] Busch M., Papior D., Stephan H., Dünker N. (2018). Characterization of etoposide- and cisplatin-chemoresistant retinoblastoma cell lines. *Oncology Reports*.

[9] Rijal S., Fleming S., Cummings N. (2015). Inositol polyphosphate 4-phosphatase II (INPP4B) is associated with chemoresistance and poor outcome in AML. *Blood*.

[10] Tang W., Yang L., Yang T. (2019). INPP4B inhibits cell proliferation, invasion and chemoresistance in human hepatocellular carcinoma. *OncoTargets and Therapy*.

[11] Agoulnik I. U., Hodgson M. C., Bowden W. A., Ittmann M. M. (2011). INPP4B: the new kid on the PI3K block. *Oncotarget*.

[12] Norris F. A., Atkins R. C., Majerus P. W. (1997). The cDNA cloning and characterization of inositol polyphosphate 4-phosphatase type II. *Journal of Biological Chemistry*.

[13] Kofuji S., Kimura H., Nakanishi H. (2015). INPP4B is a PtdIns(3,4,5)P3 phosphatase that can act as a tumor suppressor. *Cancer Discovery*.

[14] Bunney T. D., Katan M. (2010). Phosphoinositide signalling in cancer: beyond PI3K and PTEN. *Nature Reviews Cancer*.

[15] McCubrey J. A., Abrams S. L., Stadelman K. (2010). Targeting signal transduction pathways to eliminate chemotherapeutic drug resistance and cancer stem cells. *Advances in Enzyme Regulation*.

[16] Miao B., Skidan I., Yang J. (2010). Small molecule inhibition of phosphatidylinositol-3,4,5-triphosphate (PIP3) binding to pleckstrin homology domains. *Proceedings of the National Academy of Sciences*.

[17] Hamila S. A., Ooms L. M., Rodgers S. J., Mitchell C. A. (2021). The INPP4B paradox: like PTEN, but different. *Advances in biological regulation*.

[18] Alessi D. R., Andjelkovic M., Caudwell B. (1996). Mechanism of activation of protein kinase B by insulin and IGF-1. *The EMBO Journal*.

[19] Dangelmaier C., Manne B. K., Liverani E., Jin J., Bray P., Kunapuli S. P. (2014). PDK1 selectively phosphorylates Thr(308) on Akt and contributes to human platelet functional responses. *Thrombosis and Haemostasis*.

[20] Tsuchiya A., Kanno T., Nishizaki T. (2014). PI3 kinase directly phosphorylates Akt1/2 at Ser473/474 in the insulin signal transduction pathway. *Journal of Endocrinology*.

[21] Chen Y., Sun Z., Qi M. (2018). INPP 4B restrains cell proliferation and metastasis via regulation of the PI 3K/AKT/SGK pathway. *Journal of Cellular and Molecular Medicine*.

[22] Bruhn M. A., Pearson R. B., Hannan R. D., Sheppard K. E. (2010). Second AKT: the rise of SGK in cancer signalling. *Growth Factors*.

[23] Ferron M., Boudiffa M., Arsenault M. (2011). Inositol polyphosphate 4-phosphatase B as a regulator of bone mass in mice and humans. *Cell Metabolism*.

[24] Hodgson M. C., Shao L. j., Frolov A. (2011). Decreased expression and androgen regulation of the tumor suppressor gene INPP4B in prostate cancer. *Cancer Research*.

[25] Liu H., Paddock M. N., Wang H. (2020). The INPP4B tumor suppressor modulates EGFR trafficking and promotes triple-negative breast cancer. *Cancer Discovery*.

[26] Fedele C. G., Ooms L. M., Ho M. (2010). Inositol polyphosphate 4-phosphatase II regulates PI3K/Akt signaling and is lost in human basal-like breast cancers. *Proceedings of the National Academy of Sciences*.

[27] Gewinner C., Wang Z. C., Richardson A. (2009). Evidence that inositol polyphosphate 4-phosphatase type II is a tumor suppressor that inhibits PI3K signaling. *Cancer Cell*.

[28] Wu Y., Wang X., Lu Y. (2021). INPP4B exerts a dual role in gastric cancer progression and prognosis. *Journal of Cancer*.

[29] Wu Y., Meng D., Xu X. (2021). Expression and functional characterization of INPP4B in gallbladder cancer patients and gallbladder cancer cells. *BMC Cancer*.

[30] Li Chew C., Lunardi A., Gulluni F. (2015). In vivo role of INPP4B in tumor and metastasis suppression through regulation of PI3K–AKT signaling at e. *Cancer Discovery*.

[31] Wang Y., Chen L., Li Q. (2021). Inositol polyphosphate 4-phosphatase type II is a tumor suppressor in multiple myeloma. *Frontiers in Oncology*.

[32] Dzneladze I., He R., Woolley J. F. (2015). INPP4B overexpression is associated with poor clinical outcome and therapy resistance in acute myeloid leukemia. *Leukemia*.

[33] Chi M. N., Guo S. T., Wilmott J. S. (2015). INPP4B is upregulated and functions as an oncogenic driver through SGK3 in a subset of melanomas. *Oncotarget*.

[34] Guo S. T., Chi M. N., Yang R. H. (2016). INPP4B is an oncogenic regulator in human colon cancer. *Oncogene*.

[35] Woolley J. F., Dzneladze I., Salmena L. (2015). Phosphoinositide signaling in cancer: INPP4B Akt(s) out. *Trends in Molecular Medicine*.

[36] Reid T. W., Albert D. M., Rabson A. S. (1974). Characteristics of an established cell line of retinoblastoma. *Journal of the National Cancer InstituteJournal of the National Cancer Institute*.

[37] Griegel S., Hong C., Frötschl R. (1990). Newly established human retinoblastoma cell lines exhibit an “immortalized” but not an invasive phenotype in vitro. *International Journal of Cancer*.

[38] Busch M., Philippeit C., Weise A., Dünker N. (2015). Re-characterization of established human retinoblastoma cell lines. *Histochemistry and Cell Biology*.

[39] Campeau E., Ruhl V. E., Rodier F. (2009). A versatile viral system for expression and depletion of proteins in mammalian cells. *PLoS One*.

[40] Hartmann L., Neveling K., Borkens S. (2010). Correct mRNA processing at a mutant TT splice donor in FANCC ameliorates the clinical phenotype in patients and is enhanced by delivery of suppressor U1 snRNAs. *The American Journal of Human Genetics*.

[41] Busch M., Große-Kreul J., Wirtz J. J. (2017). Reduction of the tumorigenic potential of human retinoblastoma cell lines by TFF1 overexpression involves p53/caspase signaling and miR-18a regulation. *International Journal of Cancer*.

[42] Weise A., Dünker N. (2013). High trefoil factor 1 (TFF1) expression in human retinoblastoma cells correlates with low growth kinetics, increased cyclin-dependent kinase (CDK) inhibitor levels and a selective down-regulation of CDK6. *Histochemistry and Cell Biology*.

[43] Zijlstra A., Mellor R., Panzarella G. (2002). A quantitative analysis of rate-limiting steps in the metastatic cascade using human-specific real-time polymerase chain reaction. *Cancer Research*.

[44] Palmer T. D., Lewis J., Zijlstra A. (2011). Quantitative analysis of cancer metastasis using an avian embryo model. *Journal of Visualized Experiments: Journal of Visualized Experiments*.

[45] Dräger O., Metz K., Busch M., Dünker N. (2022). Role of L1CAM in retinoblastoma tumorigenesis: identification of novel therapeutic targets. *Molecular oncology*.

[46] Elso C. M., Roberts L. J., Smyth G. K. (2004). Leishmaniasis host response loci (lmr1-3) modify disease severity through a Th1/Th2-independent pathway. *Genes and Immunity*.

[47] Borowicz S., van Scoyk M., Avasarala S. (2014). The soft agar colony formation assay. *Journal of Visualized Experiments: Journal of Visualized Experiments*.

[48] Zhai S., Liu Y., Lu X. (2019). INPP4B as A prognostic and diagnostic marker regulates cell growth of pancreatic cancer via activating AKT. *OncoTargets and Therapy*.

[49] Yang L., Ding C., Tang W. (2020). INPP4B exerts a dual function in the stemness of colorectal cancer stem-like cells through regulating Sox2 and Nanog expression. *Carcinogenesis*.

[50] Chen H., Li H., Chen Q. (2016). INPP4B reverses docetaxel resistance and epithelial-to-mesenchymal transition via the PI3K/Akt signaling pathway in prostate cancer. *Biochemical and Biophysical Research Communications*.

[51] Jin H., Yang L., Wang L. (2018). INPP4B promotes cell survival via SGK3 activation in NPM1-mutated leukemia. *Journal of Experimental & Clinical Cancer Research*.

[52] Vasudevan K. M., Barbie D. A., Davies M. A. (2009). AKT-independent signaling downstream of oncogenic PIK3CA mutations in human cancer. *Cancer Cell*.

[53] Gasser J. A., Inuzuka H., Lau A. W., Wei W., Beroukhim R., Toker A. (2014). SGK3 mediates INPP4B-dependent PI3K signaling in breast cancer. *Molecular Cell*.

[54] Dickreuter E., Cordes N. (2017). The cancer cell adhesion resistome: mechanisms, targeting and translational approaches. *Biological Chemistry*.

[55] Glaser J., Neumann M. H. D., Mei Q. (2014). Macrophage capping protein CapG is a putative oncogene involved in migration and invasiveness in ovarian carcinoma. *BioMed Research International*.

[56] Huang S., Chi Y., Qin Y. (2018). CAPG enhances breast cancer metastasis by competing with PRMT5 to modulate STC-1 transcription. *Theranostics*.

[57] Prescher N., Hänsch S., Knobbe-Thomsen C. B., Stühler K., Poschmann G. (2021). The migration behavior of human glioblastoma cells is influenced by the redox-sensitive human macrophage capping protein CAPG. *Free Radical Biology and Medicine*.

[58] Chi Y., Xue J., Huang S. (2019). CapG promotes resistance to paclitaxel in breast cancer through transactivation of PIK3R1/P50. *Theranostics*.

[59] Zhou Q., Fu Q., Shaya M., Kugeluke Y., Li S., Dilimulati Y. (2022). Knockdown of circ_0055412 promotes cisplatin sensitivity of glioma cells through modulation of CAPG and Wnt/*β*-catenin signaling pathway. *CNS Neuroscience and Therapeutics*.

[60] Lang Z., Chen Y., Zhu H. (2019). Prognostic and clinicopathological significance of CapG in various cancers: evidence from a meta-analysis. *Pathology, Research & Practice*.

[61] Tsai T.-J., Chao W.-Y., Chen C.-C., Chen Y.-J., Lin C.-Y., Lee Y.-R. (2018). Gelsolin-like actin-capping protein (CapG) overexpression in the cytoplasm of human hepatocellular carcinoma, associated with cellular invasion, migration and tumor prognosis. *Anticancer Research*.

[62] Jiang S., Yang Y., Zhang Y. (2022). Overexpression of CAPG is associated with poor prognosis and immunosuppressive cell infiltration in ovarian cancer. *Disease Markers*.

[63] Zhou Y., Wang X., Zhang W. (2021). The immune-related gene HCST as a novel biomarker for the diagnosis and prognosis of clear cell renal cell carcinoma. *Frontiers in Oncology*.

[64] Qi X., Qi C., Wu T., Hu Y. (2020). CSF1R and HCST: novel candidate biomarkers predicting the response to immunotherapy in non-small cell lung cancer. *Technology in Cancer Research and Treatment*.

[65] Li K., Ma L., Sun Y. (2021). The immunotherapy candidate TNFSF4 may help the induction of a promising immunological response in breast carcinomas. *Scientific Reports*.

[66] Li Y., Chen Y., Miao L. (2021). Stress-induced upregulation of TNFSF4 in cancer-associated fibroblast facilitates chemoresistance of lung adenocarcinoma through inhibiting apoptosis of tumor cells. *Cancer Letters*.

[67] Chu H., Hui G., Yuan L. (2015). Identification of novel piRNAs in bladder cancer. *Cancer Letters*.

[68] Li L., Liu X., Ma X. (2019). Identification of key candidate genes and pathways in glioblastoma by integrated bioinformatical analysis. *Experimental and Therapeutic Medicine*.

[69] Chen Y.-H., Zhang T.-F., Liu Y.-Y. (2022). Identification of a 5-gene-risk score model for predicting luminal A-invasive lobular breast cancer survival. *Genetica*.

[70] Li C., Chan J., Haeseleer F. (2009). Structural insights into Ca2+-dependent regulation of inositol 1,4,5-trisphosphate receptors by CaBP1. *Journal of Biological Chemistry*.

[71] Pidugu V. K., Pidugu H. B., Wu M.-M., Liu C.-J., Lee T.-C. (2019). Emerging functions of human IFIT proteins in cancer. *Frontiers in Molecular Biosciences*.

[72] Tan X. F., Chen Q., Hua S. H., Yip G. W. (2021). Roles of interferon induced protein with tetratricopeptide repeats (IFIT) family in cancer. *Current Medicinal Chemistry*.

[73] Wang M., Wang W., Ding J., Wang J., Zhang J. (2020). Downregulation of Rab17 promotes cell proliferation and invasion in non-small cell lung cancer through STAT3/HIF-1*α*/VEGF signaling. *Thoracic cancer*.

[74] Liu Y., Ding Y., Nie Y., Yang M. (2020). EMP1 promotes the proliferation and invasion of ovarian cancer cells through activating the MAPK pathway. *OncoTargets and Therapy*.

[75] Liu S., Shi J., Wang L. (2022). Loss of EMP1 promotes the metastasis of human bladder cancer cells by promoting migration and conferring resistance to ferroptosis through activation of PPAR gamma signaling. *Free Radical Biology and Medicine*.

[76] Busch M., Klein S., Große-Kreul J. (2019). p53, miR-34a and EMP1-newly identified targets of TFF3 signaling in Y79 retinoblastoma cells. *International Journal of Molecular Sciences*.

