# Supplementary material for: Tumor Suppressor Role of INPP4B in Chemoresistant Retinoblastoma
Source: J Oncol. 2023 Mar 8;2023:2270097. doi: 10.1155/2023/2270097 (PMC10042642; doi:10.1155/2023/2270097)
Supplement: Supplementary Materials — Supplementary Figure 1: Quantification of Western blot analyses of AKT/p-AKT (A+B) and SGK3/p-SGK3 (C+D) expression levels after INPP4B overexpression in etoposide resistant (Etop) RB cell lines. ß-actin was used as a loading control. Values are means of at least three independent experiments ± SEM. ns p > 0.05 statistical differences compared to the control group calculated by Students t-test. [file 2270097.f1.docx]

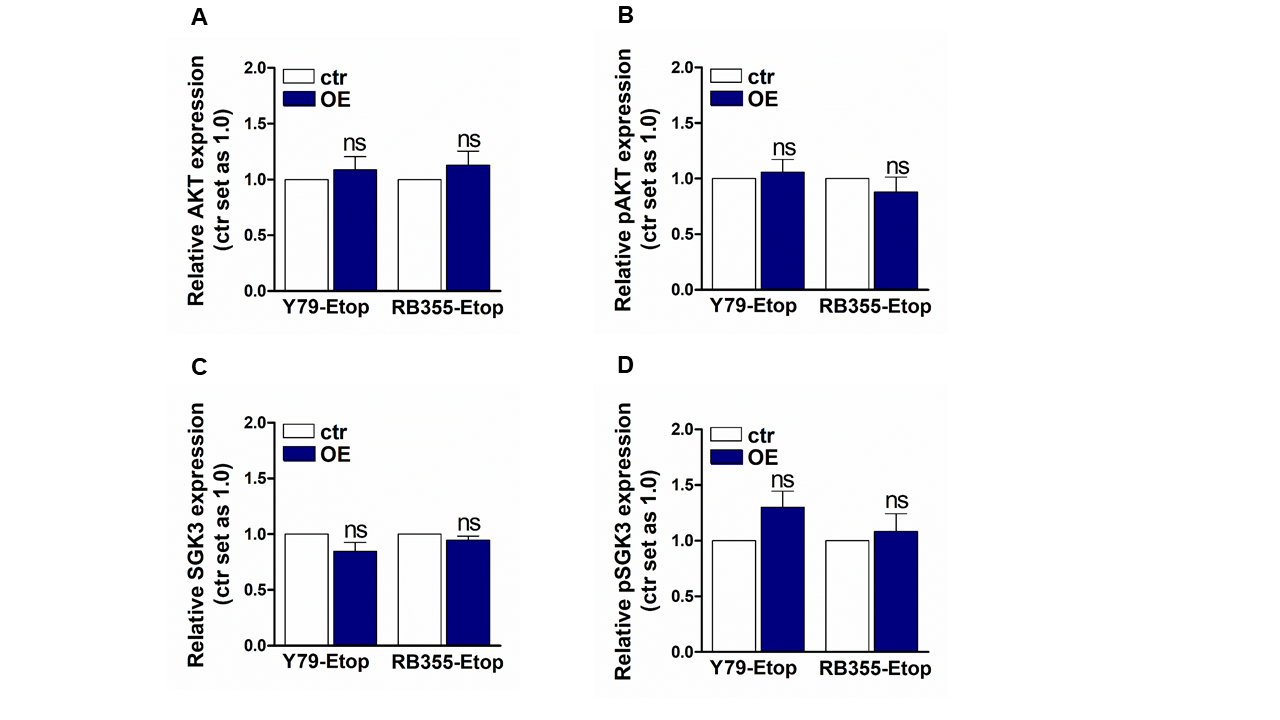


**Supplementary Figure 1.** Quantification of Western blot analyses of AKT/p-AKT (**A+B**) and SGK3/p-SGK3 (**C+D**) expression levels after INPP4B overexpression in etoposide resistant (Etop) RB cell lines. ß-actin was used as a loading control. Values are means of at least three independent experiments ± SEM. ns p>0.05 statistical differences compared to the control group calculated by Students t-test.
